# Tailoring biomaterials and applications targeting tumor-associated macrophages in cancers

**DOI:** 10.3389/fimmu.2022.1049164

**Published:** 2022-11-11

**Authors:** Fangqi Jing, Xiaowei Liu, Xiaoxuan Chen, Fanglong Wu, Qinghong Gao

**Affiliations:** ^1^ Department of Oral and Maxillofacial Surgery, West China Hospital of Stomatology, Sichuan University, Chengdu, China; ^2^ State Key Laboratory of Oral Diseases, West China College of Stomatology, Sichuan University, Chengdu, China; ^3^ Department of Prosthodontics, West China Hospital of Stomatology, Sichuan University, Chengdu, China; ^4^ State Key Laboratory of Oral Diseases, National Center of Stomatology, National Clinical Research Center for Oral Diseases, Frontier Innovation Center for Dental Medicine Plus, West China Hospital of Stomatology, Sichuan University, Chengdu, China

**Keywords:** tumor-associated macrophages, targeted therapy, cancer, crosstalk, biomaterials

## Abstract

Tumor-associated macrophages (TAMs) play a critical role in supporting tumor growth and metastasis, taming host immunosurveillance, and augmenting therapeutic resistance. As the current treatment paradigms for cancers are generally insufficient to exterminate cancer cells, anti-cancer therapeutic strategies targeting TAMs have been developed. Since TAMs are highly heterogeneous and the pro-tumoral functions are mediated by phenotypes with canonical surface markers, TAM-associated materials exert anti-tumor functions by either inhibiting polarization to the pro-tumoral phenotype or decreasing the abundance of TAMs. Furthermore, TAMs in association with the immunosuppressive tumor microenvironment (TME) and tumor immunity have been extensively exploited in mounting evidence, and could act as carriers or accessory cells of anti-tumor biomaterials. Recently, a variety of TAM-based materials with the capacity to target and eliminate cancer cells have been increasingly developed for basic research and clinical practice. As various TAM-based biomaterials, including antibodies, nanoparticles, RNAs, etc., have been shown to have potential anti-tumor effects reversing the TME, in this review, we systematically summarize the current studies to fully interpret the specific properties and various effects of TAM-related biomaterials, highlighting the potential clinical applications of targeting the crosstalk among TAMs, tumor cells, and immune cells in anti-cancer therapy.

## Introduction

It has been illustrated that tumor initiation and progression are not only related to the genomic changes of cancerous cells, but are also affected by the tumor microenvironment (TME) (1, 2). Tumor associated macrophages (TAMs), as a key regulator in TMEs, are made up of a mix of tissue resident and exudative macrophages in varying proportions based on the type, location, and stage of the tumor (3). Typically, TAMs can be designated as M1- and M2-polarized macrophages. M1 macrophages produce pro-inflammatory cytokines, including interleukin‐1β (IL‐1β), IL‐6, IL‐12, tumor necrosis factor‐α (TNF‐α), and interferon‐γ (IFN‐γ), etc., which activate host immune responses against microbes and viruses, subsequently leading to the suppression of tumor progression (4). While M2 macrophages secrete anti‐inflammatory cytokines, including IL‐10, IL‐13, and transforming growth factor‐β (TGF‐β), exerting the promotion of cancer occurrence and development (5). However, there are still some situations where M2 macrophages might be an inhibitor in tumor progression. For instance, Rakaee et al. provided evidence showing that high level of CD204^+^/CD68^+^ M2 macrophages would be an independent positive prognostic marker of prolonged survival in lung cancer (6), indicating the exact function of the TAMs highly depends on cellular phenotype. To support this notion, in our preliminary study, we found that oral cancer metastasis in clodronate-treated mice was not significantly reduced by M1/2 macrophage reduction (7), suggesting that TAMs with specific surface markers, instead of the board M1/2 macrophages, exert various cellular functions in cancers. Although the controversy of TAMs subtypes in tumor biological behaviors, TAMs are still extensively studied and regarded as a great potential target. Importantly, various TAMs-related materials have been created for anti-tumor therapy not only in the basic research but also in the pre-clinical settings.

Biological materials based on TAMs have recently been divided into three main aspects as follows: materials targeting TAMs directly, and those targeting TAMs indirectly through cancer cells and through immune cells (8, 9). An increasing number of studies of biological materials targeting TAMs have focused on experimental and pre-clinical anti-tumor approaches with encouraging signs; however, there are still a number of challenges to overcome before they can be employed in clinical practice. For instance, materials aiming at TAMs polarization might be oversimplified and problematic, as TAMs cannot be readily split into M1/M2 macrophages due to the existence of more nuanced phenotypes (10, 11). The cellular marker cluster of differentiation (CD68) has been widely used as a pan-macrophage marker in most studies; however, it has been reported that CD68 occasionally expressed in dendric cells, stromal cells, even cancer cells (12), indicating that any biological materials targeting CD68^+^ TAMs might be off-target. Furthermore, there are still some obstacles to the optimization of biocompatibility and efficacy in the application of nanomaterials targeting TAMs. Thus, to comprehensively understand the current progress of TAMs based on materials, in this review, we systematically summarized the design and application of materials based on TAMs from various routes and targets, providing new insights for anti-cancer therapeutic avenues.

## Materials targeting tumor-associated macrophages directly

### TAM phenotypic heterogeneity and its relationship with materials application

TAMs are highly heterogeneous stromal cells, and these distinct phenotypic characteristics are well established by techniques including immunohistochemistry, flow cytometry, single-cell sequencing, etc. Recently, it has been proposed that tissue macrophages arise from not only blood monocytes, but also the embryonic precursors deriving from the yolk sac and/or fetal liver (13, 14). The differentiated cell type’s chromatin landscape, among other epigenomic traits, represents the macrophage developmental origins (15), indicating that epigenetic modification and ontogeny can influence its identity development and thus dictate phenotypic heterogeneity. Furthermore, the TME in different cancers could also significantly alter macrophages phenotypes in distinct anatomical regions. For instance, TAMs are formed of a heterogeneous population of macrophages in hepatocellular carcinoma and breast cancer (16, 17). Interestingly, even in the same TME, the majority of the TAMs population differs in phenotype, which is related to the distances between cells, and systemic toxicity might result if all types of TAMs are targeted. To support this, using mass cytometry with extensive antibody panels, Chevrier et al. found that there were 17 unique macrophage phenotypes in the TME of human renal cell carcinoma, and even that the same type of macrophage not only expressed the CD169 (as an anti-tumorigenic marker), but also co-expressed with pro-tumorigenic markers including CD163, CD206, etc. (10), suggesting that the application of targeting materials should focus on, or be aware of, the most important subsets and paradoxical behaviors to optimize the therapeutic efficacy. Another potential hypothesis for the different phenotypic heterogeneity in the same type of tumor is that the environment caused by distinct sites might modulate macrophage phenotypes. For instance, in gastric cancer, Huang et al. provided data showing that CD68^+^IRF8^+^ macrophages dominated in the closest sites to the cancer cells, while the CD68^+^CD163^+^CD206^+^ macrophages dominated in the furthest sites (18), demonstrating the macrophages marker expression differences in tumor areas. Due to heterogeneity, TAMs release different mediators: either anti-tumor, including IL-6, IL-1β, chemokine (C-C motif) ligand 2 (CCL2), and TNF, or pro-tumor, including IL-10, TGF-β, vascular endothelial growth factor (VEGF), platelet-derived growth factor (PDGF), CCL17, CCL22, and CCL24 (19, 20). By using the monoclonal and specific antibodies for cellular markers, various cytokines and transcriptional profiles have been utilized for targeting TAMs (21). In summary, the phenotypic heterogeneity of TAMs depends on cellular origins, cancer type, and cellular distribution in the TME, etc., and with the discovery of more specific markers, we will be able to more accurately identify the subpopulations and develop the targeting materials for anti-cancer therapy.

### TAM co-culture systems and their relationship with materials application

**Figure 1:**
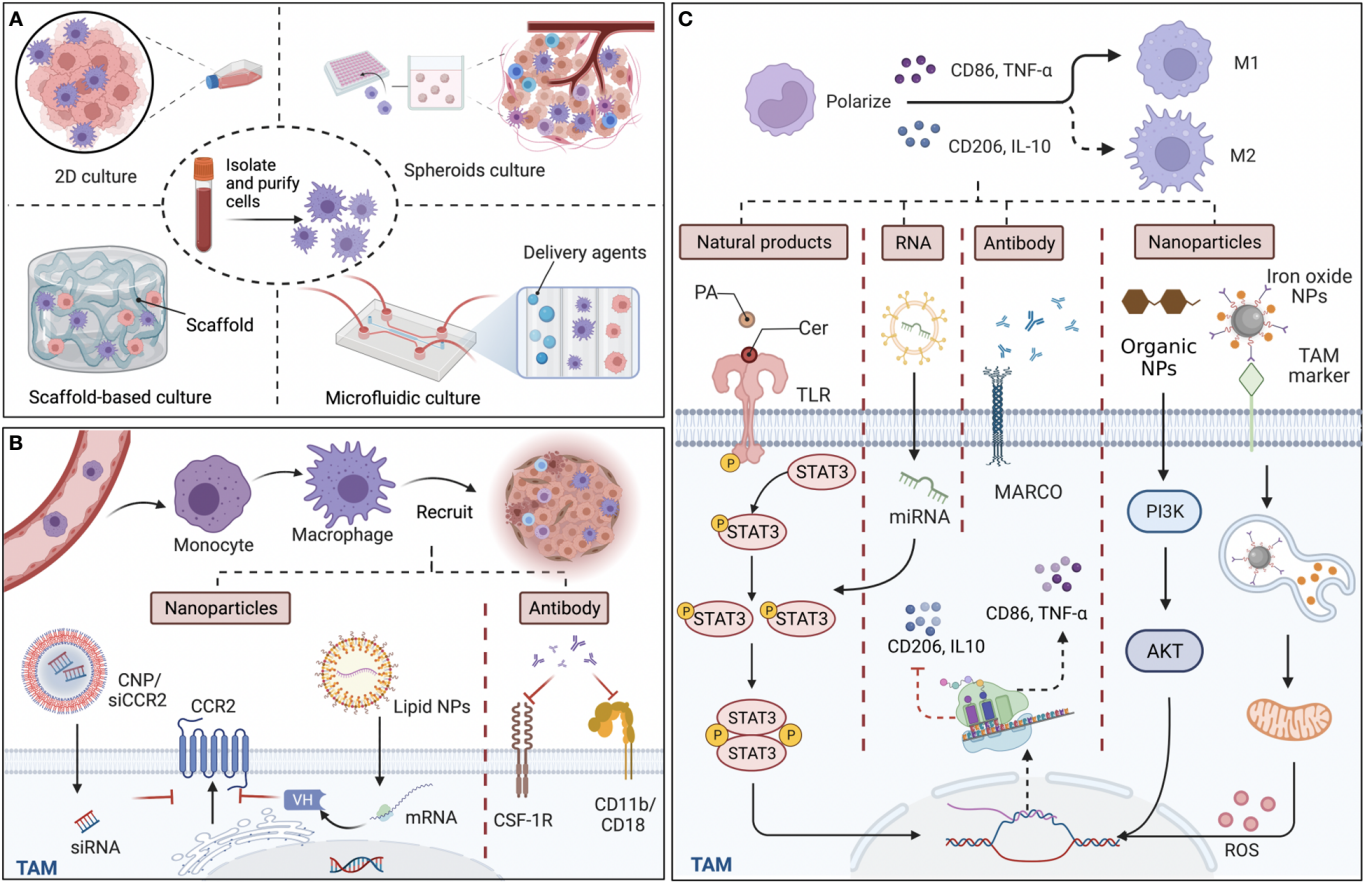
Culture models *in vitro* for tumor-associated macrophages (TAMs) and targeted approaches for TAM recruitment and polarization. **(A)** The structure of main TAM co-culture systems. Compared with two-dimensional (2D) cultures, which traditionally cultivate macrophages and cancer cells on a plane, 3D culture models including spheroids, scaffold-based models, and microfluidic-based models are more accurate in reproducing cell and tissue physiology. **(B)** TAMs derive from monocytes and recruit into tumor sites guided by various chemokines and cytokines. By targeting chemokine (C-C motif) receptor 2 (CCR2), colony-stimulating factor 1 receptor (CSF-1R), and CD11b/CD18, nanoparticles (NPs), including siRNA-coated NPs (CNP/siCCR2) and lipid NPs, and antibodies can effectively reduce TAM infiltration and reshape immunosuppressive tumor microenvironments. **(C)** Since M1 macrophages are typically identified by the secretion of surface markers CD86 and tumor necrosis factor-α (TNF-α), while M2 macrophages are identified by CD206 and interleukin‐10 (IL‐10), the main mechanism of the various materials that affect the polarization of TAMs is interference with the expression of the relevant markers. Some natural ingredients, such as ceramide (Cer) and palmitic acid (PA), could act as agonists of toll-like receptors (TLRs), efficiently modulating the STAT3 signaling pathways and polarizing TAMs to the M1 phenotype. Regarding artificial ingredients, exosomal miRNA, which affects the diverse signaling pathways for the expression of markers, monoclonal antibodies (mcAbs) against MARCO receptor, which is expressed by M2 macrophages can repolarize M2 back to TAMs, and NPs, modulate related signaling pathways and reactive oxygen species (ROS) release, exhibiting with efficacy in cancer therapy. Back arrows: promotion; Red “T” arrows: inhibition.

To monitor the biological behavior of TAMs, co-culture systems, which are necessary in recreating TMEs, could provide a promising human *in vivo*-like tissue model, including two-dimensional (2D) and three‐dimensional (3D) cell cultures (**Figure 1A**). *In vitro* 2D cell cultures, such as Transwell inserts with Matrigel, have been widely used to explore the polarization and pertinent signaling pathways of TAMs *in vitro* (22). However, limited by physical structure and components, existing 2D models might remodel cells and their internal cytoskeleton, and affect cell arrangements on a flat substrate (23), making the exploration of cell performance and the simulation of natural environments *in vivo* difficult. Thus, 3D co-culture systems have arisen to mimic the situation *in vivo*, in which the spatial organization of cells is more reliable for the physiological relevance of experiments. At present, the types of 3D co-culture models related to TAMs can mainly be classified into spheroids, scaffold-based models, and microfluidic-based 3D models (**Figure 1A**). The 3D multi-cellular spheroid model, using the ‘hanging drop’ approach, or aggregate cultures with a matrix construction including heterogeneous populations of cells could be devised with hypoxia and necrotic patches to imitate tumor features *in vivo* (24, 25), leading to better comprehension of TAM performance as influenced by the reactive oxygen species (ROS) and hypoxic region in the TME. Regarding scaffold-based models, a large number of either organic or inorganic matrices and scaffolds have been employed to mimic the extracellular matrix due to good biocompatibility. For instance, Matrigel is composed of a reconstituted basement membrane extract (BME) secreted by a mouse sarcoma, and the natural scaffolds consists of purified proteins such as type I collagen, while artificial scaffolds are made up of polyethylene glycol (PEG)-based hydrogels and synthetic alternatives to Matrigel (26). Additionally, microfluidics, with high spatial controllability, can support short-term culture, and are usually designed with a different number of chambers and lateral channels. For example, utilizing the adjacent gel channels in a microfluidic device to explore paracrine signals, Huang et al. found that the macrophages invaded the gels composed of type I collagen and Matrigel, and transformed into distinct phenotypes when cultured next to the cancer cells (27). Together, although the 3D *in vitro* models have been widely employed to mimic *in vivo* settings for more closely analyzing the cell-to-cell crosstalk and the organoid, etc., the inner cells in the sphere models might not be flourished by angiological system like them in human tumors. In the spheroids, cells locating in different zones exhibit different proliferation, and the tumor cells in the core exhibited quiescent due to the limited oxygen and nutrient delivery (28). Unlike this, the inner cells in human tumors could be flourished by vascular and lymphatic vessels, even the specific vasculogenic mimicry (VM) which was defined as a fluid conducting channel embedded in extracellular matrix to feed tumor cells (29). Of note, the majority of macrophages originate from monocytes in blood and affiliate in the perivascular lesions; thus, one of the challenges for 3D co-culture systems for macrophages is to create microcirculation in the 3D culture models and locate the macrophages beside extravascular sites.

### TAM recruitment and its relationship with materials application

Monocytes and monocyte-related myeloid-derived suppressor cells (M-MDSCs) are the main precursors of TAMs, which are recruited to the tumor site under the guidance of a variety of chemical attractants and influence tumor progression; thus, various materials targeting TAM recruitment have been created for anti-cancer therapy (**Figure 1B**). Since the process is influenced by chemokines, such as CCL2, colony-stimulating factor 1 (CSF-1), chemokine (C-X-C motif) ligand 12 (CXCL12), etc., and cytokines, including IL-6, IL-8, IL-34, and members of the VEGF family, blocking the related signaling pathways with nanoparticles (NPs) or specific antibodies is an effective way to affect TAM recruitment from both an epigenetic and molecular level. Taking CCL2-CCR2 signaling pathways as an example, Shen et al. found that prepared siRNA-coated NPs (CNP/siCCR2) effectively inhibited CCR2 expression and blocked the recruitment of monocytes to the tumor site, leading to reduced tumor metastasis (30). Despite the encapsulation of direct effectors, lipid NPs could also load mRNA, which could translate a single-domain antibody (V_H_) as bispecific CCL2/CCL5 inhibitors (BisCCL2/5i), reversing the infiltration of TAMs in the TME (31). In another blocking approach, monoclonal antibodies (mcAbs) targeting signals in TAM recruitment have also been developed as anti-tumor therapy by binding to and neutralizing the surface markers, and then modulating TAM recruitment with the suppression of adhesion and migration to the vascular endothelium and chemotaxis, including anti-CSF-1R mcAbs (32) and neutralizing CD11b mcAbs, which could also act as adjuvants to radiotherapy (33). Therefore, targeting the specific signaling pathways of monocytes can effectively inhibit TAM recruitment, reduce TAM infiltration in solid tumors, and reshape the immunosuppressive TME, while the protection of monocytes that derived to macrophages in normal tissues is worth noting in the process. To support this, Mehta et al. provided data demonstrating that anti-CSF-1R antibodies influenced monocyte/macrophage migration in triplenegative breast cancer (34). In summary, compared with injecting antibodies themselves, mRNA-loaded NPs, which allow the generation of immunotherapeutic proteins in the TAM nucleus and cytoplasm, are equipped with a specific signaling peptide at the N-terminal end of BisCCL2/5i and secrete to the TME effectively, thus taking effect at lower doses and reducing possible systemic toxicity. Furthermore, to maximize the efficacy of agents, which are widely distributed, preferential aggregation in myeloid cells around bone marrow and peripheral blood is the potential area of inquiry taking advantage of the properties of materials.

### TAM polarization and its relationship with materials application

Characterized by different surface markers, secretions, and functions, the polarization state of TAMs is not constant as a result of plasticity, which transforms towards the M1 phenotype for pro-inflammation and anti-tumor effects, while alternatively to M2 exerting anti-inflammation and pro-tumor functions. At present, the strategy of targeting TAM polarization is primarily accomplished by regulating signals with agonists and inhibitors, etc. (**Table 1**). With regard to agent type, biological materials are mainly composed of natural and artificial ingredients, including RNA, antibodies, NPs, etc. (**Figure 1C**). For instance, lipids in natural ceramide (Cer) and palmitic acid (PA) could bind to TLR (toll-like receptor) and cause M2 phenotype repolarization, subsequently inhibiting human colon cancer invasion (39). Since these natural lipids are non-toxic, highly concentrated *in vivo*, and easily isolated from plasma membrane, powerful properties might be further explored in blocking TAM polarization for anti-cancer therapy. Artificial materials such as exosomal miRNAs, which could influence the production of target mRNAs by binding to their 3′untranslated regions (3′UTRs), utilize transcription factors including PPARs, STAT3/6, etc., and induce TAM repolarization at the transcriptional level. For instance, Ying et al. showed that exosomal miR-222-3p promoted M2 macrophage polarization by the SOCS3/STAT3 pathway in ovarian cancer (50). Furthermore, mcAbs based on blocking TAM polarization could interact with related receptors, directly affecting tumor progression. For example, MARCO expressed by M2 macrophages and the injection of its specific antibody (ED31) decreased tumor growth and metastasis *in vivo* by polarizing TAMs to the M1 subtype (51). Importantly, organic NPs, RP6530 (C_23_H_18_FN_5_O_2_), a novel PI3Kδ/γ inhibitor with nanomolar inhibitory potency, suppressed tumor growth by downregulating the limiting glycolytic enzyme PKM2 and repolarizing M2 (44), indicating that kinases in signaling pathways related to macrophage phenotypic polarization can also be targeted. Different from influencing TAMs themselves, inorganic NPs, such as ferumoxytol and calcium carbonate NPs, can mediate the repolarization of TAMs by regulating the ROS and acidity of the TME. For instance, Zanganeh et al. found that, with the increase in M1, ferumoxytol significantly inhibited the growth of subcutaneous adenocarcinoma in mice (52). Notably, due to the limitations of inorganic materials’ own properties on the human body in that iron over-exposure might increase the incidence of cancer by increasing oxidative stress and DNA damage, the application needs to pay strict attention to the dosage and timing. On the whole, therapeutic strategies targeting TAM polarization processes mainly include the modulation of gene expression, surface receptors, and the TME, among which organic NPs targeting enzymes or signaling pathways might be more effective without reliance on FcR-like antibodies.

**Table 1:** Application of targeting materials for TAM polarization in cancers.

Tumor	Impact on targets	Agent	Carries	Applications	Effects	Ref.
BC	STAT3 and NF‐κB inhibitor	HA‐coated PeiPLGA‐MTX	NPs	*In vivo*	Repolarize M2	(35)
BC, MEL	TLR4 agonist	Paclitaxel	N/A	*In vivo* and vitro	Guide TAMs to M1	(36)
BCL, CC	TLR9 agonist	IMO-2125	N/A	*In vivo*	Increase M1	(37)
CC	CSF-1R inhibitor and CD40 agonist	N/A	N/A	*In vivo* and vitro	Repolarize M2	(38)
CC	IL-10/STAT3/NF-κB signal inhibitor	Cer; PA	N/A	*In vitro*	Block M2 polarization	(39)
CC	PI3K inhibitor	LY294002	N/A	*In vitro*	Modulate M2 polarization	(40)
GBM, MEL, OC	IRF5/IKKβ antagonist	IVT-mRNA	NPs	*In vivo*	Repolarize M2	(41)
HCC	HGF and MIF inhibitor	miR-144/miR-451a	N/A	*In vitro*	Increase M1	(42)
HCC	RelB/p52 antagonist	Baicalin	N/A	*In vivo* and vitro	Induce TAMs to M1	(43)
HL	PI3Kδ/γ inhibitor	RP6530	N/A	*In vivo* and vitro	Switching M1-like reprogramming	(44)
HNSCC	TLR7 agonist	1V270	N/A	*In vivo*	Increase ratio of M1/M2	(45)
MEL	CSF-1R inhibitor	Anti-CSF-1R siRNA (siCD115)32	M2NPs	*In vivo*	Modulate M2 polarization	(46)
PC	Boost IL-12, TLR agonist	ZA	N/A	*In vitro*	Polarize to M1 TAMs	(47)
PDAC	HLA-DR, CD40, CCR7 agonist; CD163, CD206 inhibitor	Gemcitabine	N/A	In clinic trial	Modulate M1 polarization	(48)
SAR	TLR4 agonist	Cationic polymers	N/A	*In vivo* and vitro	Reverse TAMs polarization	(49)

BC, breast cancer; BCL, B-cell lymphoma; CA, corosolic acid; Cer, ceramide; CC, colon cancer; CSF-1R, colony-stimulating factor 1 receptor; GBM, glioblastoma; HA, hyaluronic acid; HCC, hepatocellular carcinoma; HDAC, histone deacetylase; HGF, hepatocyte growth factor; HL, Hodgkin lymphoma; HNSCC, head and neck squamous cell carcinoma; IKKβ, a kinase that phosphorylates and activates IRF5; IRF5, Interferon Regulatory Factor 5; IVT, *In vitro*-transcribed; LCL, long-circulating liposomes; M2NPs, M2-like TAM dual-targeting nanoparticles; MEL, melanoma; MIF, migration inhibitory factor; miRNA, microRNA; N/A, not available; NPs, nanoparticles; NSCLC, non-small-cell lung cancer; OC, ovarian cancer; OSCC, oral squamous cell carcinoma; PA, palmitic acid; PC, prostate cancer; PDAC, pancreatic ductal adenocarcinoma; PeiPLGA‐MTX, polyethyleneimine poly(lactic‐co‐glycolic acid) carrying methotrexate; SAR, sarcoma; siRNA, small interfering RNA; TAMs, tumor-associated macrophages; TLR, toll-like receptor; ZA, zoledronic acid.

## Materials based on tumor-associated macrophages for targeting cancer cells

### The application of TAM-related antibodies for targeting cancer cells

**Figure 2:**
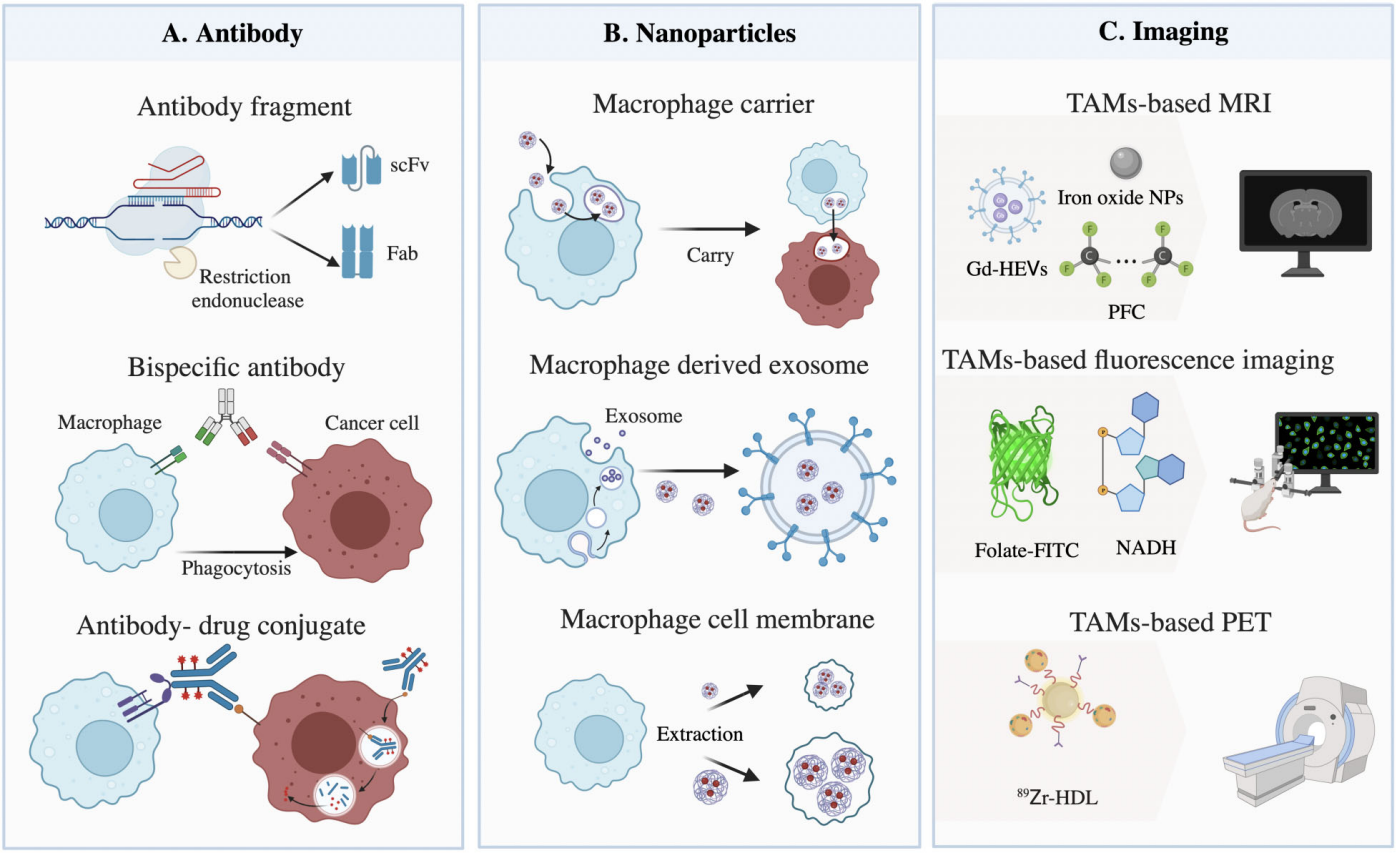
Materials based on tumor-associated macrophages (TAMs) for targeting cancer cells. **(A)** Illustration of restructured antibody targeting TAMs and cancer cells. The method mainly consists of three parts: antibody fragments, bispecific antibodies, and antibody-drug conjugates. **(B)** Macrophages, exosomes, and cell membranes act as nanoparticle carriers to target tumors and induce phagocytosis. **(C)** Materials used for TAM-based imaging, composed of gadolinium (Gd) with extracellular vesicles (Gd-HEVs), iron oxide nanoparticles (NPs), and perfluorocarbon compounds (PFCs) for magnetic resonance imaging (MRI), folate-conjugated fluorescein isothiocyanate (folate-FITC) and nicotinamide adenine dinucleotide (NADH) for fluorescence imaging, and ^89^Zr-high-density lipoprotein (HDL) for positron emission tomography (PET).

Therapeutic antibodies can stimulate immune-mediated tumor cell death by engaging innate immune cell lineages or activating complement cascades once they have been bound to cell surface antigens. As immune effectors, macrophages can not only express antibody fragment crystallizable receptors (FcR), but also crosstalk with tumor cells through the efficiency of various antibodies as they could directly execute antibody-dependent cell-mediated cytotoxicity (ADCC) and antibody-dependent cellular phagocytosis (ADCP), as well as complement-dependent cytotoxicity (CDC). TAMs in the TME are crucial to increase the effectiveness of antibody-mediated cancer immunotherapy, and the functions of TAMs have been proven to contribute to mcAbs’ effectiveness *in vitro*, *in vivo*, and in clinical trials (**Table 2**). According to the structural and functional differences, antibodies can be divided into IgA, IgD, IgE, IgG, and IgM, among which IgG is the most commonly used mcAb, exerting therapeutic actions by attracting cells with FcγR family members, including FcγR I, FcγR II, and FcγR III, or blocking the molecular signal (73). The interaction between FcγR on macrophages and immune complexes leads to enhanced phagocytosis and the release of superoxide, TNF, tissue plasminogen activator, prostaglandins, and leukotrienes (74), which further modulates tumor development. To fully engage TAMs, improvements in binding efficacy with antibodies could be carried out by changing Fc fragments with genetic modification by splicing with overlap extension (75), and protein modification by modulating Fc regions to be fucosylation-free (76). Except for genetic and protein engineering, remodeled antibodies are applied in anti-cancer therapy, including antibody fragments, bispecific antibodies (bsAbs), and antibody-drug conjugates (ADCs) (**Figure 2A**).

**Table 2:** Antibodies based on TAMs in anti-cancer therapies.

Tumor	Agents	Antibody	Target	Macrophages	Effects of TAMs	Application	Ref.
B-CLL	Rituximab (Mabthera)	Human IgG1 mcAbs	CD20	hMDMs (M1/2-like)	ADCP	*In vitro*	(53)
BC	Trastuzumab	Humanized IgG2 mcAbs	HER2	Mice BMDM, hMDMs	ADCP	*In vivo*	(54)
BC, BL, CRC, RC	KWAR23	Human IgG bsAbs	SIRPα/CD47	Human macrophages	ADCP	*In vitro* and *vivo*	(55)
BC, CRC, epidermoid carcinoma	Clone 528	HAMAs	EGFR	THP-1 monocytes	ADCP	*In vitro* and *vivo*	(56)
BC, NSCLC	YW327.6S2	Human IgG mcAbs	Axl	Macrophages in mice	Reduce inflammatory cytokines	*In vivo*	(57)
CRC	1H9	Humanized IgG1 mcAbs	SIRPα	hMDMs	ADCP	*In vitro* and *vivo*	(58)
CRC; HCC; MEL	RMP1-14	Human anti-murine ADC	PD-1	Macrophages in mice	Deplete TAMs	*In vivo*	(59)
CRPC	Carlumab (CNTO 888)	Human IgG1κ mcAbs	CCL2	Human macrophages	Block TAMs differentiation	In clinical trial	(60)
Dt-GCT	Emactuzumab (RG7155)	Humanized IgG1 mcAbs	CSF1R	hMDMs (M1/2-like) and human macrophages	Deplete TAMs	In clinical trial	(61)
HL	SGN-30	Chimeric IgG1 mcAbs	CD30	hMDMs	ADCP	*In vitro* and *vivo*	(62)
HL, MM, NHL, RC	Clone 1F6	Human IgG1 mcAbs	CD70	hMDMs	ADCP, CDC and ADCC	*In vitro* and *vivo*	(63)
MM	Daratumumab	Human IgG1 mcAbs	CD38	hMDMs	ADCP	*In vitro* and *vivo*	(64)
MM	Elotuzumab	Humanized IgG1 mcAbs	SLAMF7	Macrophages in mice	ADCP	*In vitro* and *vivo*	(65)
MM	J6M0-mcMMAF (GSK2857916)	Humanized ADC	BCMA	hMDMs	ADCP and ADCC	*In vitro* and *vivo*	(66)
MM	XmAb5592	Humanized IgG1κ mcAbs	HM1.24	hMDMs	ADCP	*In vitro* and *vivo*	(67)
NHL	Hu5F9-G4	Humanized, IgG4 mcAbs	CD47	Human macrophages	ADCP	In clinical trial	(68)
PDA	CP-870,893	Human IgG2 mcAbs	CD40	Human macrophages	Enhance TAA presentation	In clinical trial	(69)
Solid tumors	AMG 820	Human mcAbs	CSF1R	Human macrophages	Deplete TAMs	In clinical trial	(70)
Solid tumors	Amivantamab	Human IgG1 bsAbs	EGFR/cMet	Macrophages in mice	ADCP	*In vitro* and *vivo*	(71)
Solid tumors	Carlumab	Human IgG1κ mcAbs	CCL2	Human macrophages	Deplete TAMs	In clinical trial	(72)

ADC, antibody-drug conjugate; ADCC, antibody-dependent cell-mediated cytotoxicity; ADCP, antibody-dependent cellular phagocytosis; B-CLL, B-chronic lymphocytic leukemia; BC, breast cancer; BCMA, B-cell maturation antigen; BL, Burkitt lymphoma; BMDM, bone marrow-derived macrophage; bsAbs, bispecific antibodies; CCL2, C-C chemokine ligand 2; CDC, complement-dependent cytotoxicity; CRC, colorectal cancer; CRPC, castration-resistant prostate cancer; Dt-GCT, diffuse-type giant cell tumor; HAMAs, human anti-murine antibodies; HCC, hepatocellular carcinoma; HL, Hodgkin lymphoma; hMDMs, human monocyte-derived macrophages; mcAb, monoclonal antibody; MEL, melanoma; MM, multiple myeloma; N/A, not available; NHL, non-Hodgkin lymphoma; NSCLC, non-small-cell lung cancer; PDA, pancreatic ductal adenocarcinoma; RC, renal carcinoma; TAA, tumor-associated antigen; TAMs, tumor-associated macrophages.

The molecular weight of the Fab fragment obtained by restriction endonuclease digestion is equivalent to 1/3 of the intact antibody. The molecular weight of the single-chain variable fragment (scFv) or single domain antibody (V_H_, V_L_ nanobody) prepared by genetic engineering is even smaller, composed of approximately 120 amino acids and equivalent to 1/6 or 1/12 of the intact antibody, respectively, thus improving its penetration of solid tumors (77). For instance, chimeric antigen receptor (CAR) macrophage therapy could target cancer cells based on CD3ζ, which is homologous to the FcϵRI-γ, a canonical signaling molecule for ADCP, and repolarize M2 macrophages, therefore promoting antigen-specific phagocytosis and tumor clearance *in vitro* (78). Notably, while designing, appropriate miniaturization should be carried out according to its pharmacokinetic characteristics, to avoid weakening the affinity between antibody and target antigens, and reducing the clinical effects owing to the decrease in half-life.

BsAbs are roughly classified into fragment-based bsAbs, composed of two scFvs, and Fc-based bsAbs, with Fc domains linked to Fab or scFv, which could target tumor antigens and conjunct with macrophages or other types of antigenic epitopes at the same time, enhancing binding efficacy and anti-tumor specificity. For instance, Chen et al. provided data demonstrating that bsAbs targeted FcγR and stimulated innate and adaptive responses in the TME, leading to the polarization and phagocytosis of TAMs (79). In addition, compared with Fc-based bsAbs, the former has a lower molecular weight, indicating better penetration in solid tumors and faster clearance. Moreover, catumaxomab, a trifunctional antibody (trAb), can bind three different cell types: tumor cells, T-cells, and accessory cells (80), broadening our horizons in the design of antibodies. Although this method offers great potentials, the affinity and stability of bsAbs are influenced by the properties of additional linkers, which need to be taken into consideration.

Acting as another class of highly promising antibody-based therapeutics, ADCs are composed of an antibody targeting cancer cell-specific antigens, a cytotoxic drug, and a chemical linker that connects the drug and the antibody. Cancer targets for approved ADCs vary, such as CD33 for acute myeloid leukemia (81), and HER2 for lung adenocarcinoma (82). When mentioned drugs, pyrrolobenzodiazepine (PBD) dimers, a type of anti-tumor/antibiotic natural chemical generated by actinomycetes, are the most potent class of cytotoxic drugs, followed by maytansinoids, auristatins, and calicheamicin, all of which have similar activity (83). Specifically, in order to improve macrophage phagocytosis, materials conjugated to the linkers could also be replaced by immunostimulatory drugs, including TLR agonists, scavenger receptor ligands, proinflammatory cytokines, chemokines, etc. For instance, Dela Cruz et al. showed that, compared with the unmodified antibody, an anti-HER2 antibody fused with granulocyte-macrophage colony-stimulating factor (GM-CSF), a cytokine associated with an increased expression of major histocompatibility complex class II (MHC-II) on monocytes, was more stable in the blood and more effective in activating TAMs, leading to an enhanced anti-tumor response (84). Of note, before the internalization of ADCs, extracellular enzymes released by surrounding cancer cells and TAMs could cleave diffusible drugs, resulting in surrounding ‘bystander’ cell death. Thus, antibody specificity, payload drug cytotoxicity, linker stability, and cleavage should be fully considered when designing ADCs.

### The application of nanoparticles based on TAM carriers for targeting cancer cells

NPs with a small size and diverse surface characteristics are proper materials for penetrating poorly vascularized and fibrotic tumors. At present, NPs targeting cancer cells, in mainstream design, are based on enhanced permeation and retention (EPR), due to the leaky vasculature and poor drainage of solid tumors. Recent research has revealed that most NPs enter the tumor site through endothelial cells actively instead of inter-endothelial gaps passively (85). Thus, taking advantage of preferential aggregation within macrophages after systemic delivery and the strong infiltration potential of myeloid cells, the loading of NPs into monocytes/macrophages in the blood circulation system can be employed to facilitate drug release in the tumor’s bulk. Furthermore, TAMs can cross the blood-brain barrier (BBB) and infiltrate further to the hypoxic TME, driven by oxygen gradients and signal pathways without reliance on the EPR effect (86). Studies on NPs drug delivery systems use a variety of materials, including biological carriers, viral particles, carbon nanotubes, albumin NPs, etc. (**Table 3**), among which liposomes, polymeric NPs, and iron oxide NPs are the most commonly used. Moreover, as a component of Live Cell-mediated Drug Delivery Systems (LCDDS), M1 macrophages, which can target tumors and promote inflammation in the TME, act as drug carriers with either the complete or chosen essential components, including exosomes and external cellular membranes (105) (**Figure 2B**).

**Table 3:** Nanoparticles engulfed by TAMs for anti-cancer therapy.

Type	Tumor	Diameter (nm)	Cargo	Active target	Effects	Animal model	Application	Ref.
Acetylated CMC	PCA	120	Docetaxel	N/A	Deplete macrophages	Female C57BL/6 mice	*In vivo*	(87)
Albumin NPs with dual ligands	Glioma	~135	DSF/Cu, Rego	SPARC, mannose receptors	Inhibit cancer proliferation and repolarize M2	Male Balb/c, C57BL/6, Sprague Dawley mice	*In vivo* and vitro	(88)
AuNRs	BC	~6	HS-PEG	N/A	Tumor imaging and treatment	Female Balb/C mice	*In vivo* and vitro	(89)
Calcium carbonates NPs	MEL	~100	Anti-CD47 Ab	CD47	Activate M1	Female C57BL/6 mice	*In vivo* and vitro	(90)
CNP	BC	120.9 ± 12.2–128.3 ± 18.1	siCCR2	CCR2	Decrease TAMs’ abundance	Female Balb/C mice	*In vivo* and vitro	(30)
Copper NPs	PDAC	4.9 ± 0.3	Gemcitabine	CCL2/CCR2	Inhibit TAMs recruitment	Female C57BL/6 mice	*In vivo* and vitro	(91)
CPMV	MEL	30	Photosensitizer	N/A	Target TAMs and cancer cells	N/A	*In vitro*	(92)
Exo-Ab	BC	100	Ab of CD47 and SIRPα	CD47, SIRPα	Repolarize M2	Balb/C mice	*In vivo* and vitro	(93)
HDL-based NPs	BC	10.9 ± 2.8	^89^Zr-label	CSF-1R	Imaging of TAM	Female MMTV PyMT mice	*In vivo*	(94)
Hollow MnO_2_ NPs	LC	3.4	3PO and LOX	Glycolysis	Decrease M2	B16F10-tumor-bearing mice	*In vivo* and vitro	(95)
Iron oxide NPs	BC, LC	N/A	N/A	N/A	Enhance cancer immunotherapy	Female FVB/N mice	*In vivo* and vitro	(52)
Liposomes	MEL	116 ± 3.7 – 118 ± 2.6	PD-L1 Ab, PTX/αGC	PD-1/PD-L1	Enhance anti-tumor effects	Female C57BL/6 mice	*In vivo*	(96)
Liposomes	Multiple cancer	75-100	Alendronate, doxorubicin	N/A	Modulate TAMs polarization	Female Balb/C, Sabra mice	*In vivo* and vitro	(97)
MpSi particles	BC, LC	20- 50	Nab-PTX	N/A	Inhibit tumor growth	Female Balb/C, C57BL/6 mice	*In vivo* and vitro	(98)
Nab	PDAC	N/A	PTX	TLR4	Increase M1	Female C57BL/6 mice	*In vivo* and vitro	(99)
NK-NPs	BC	85 ± 1.2	TCPP	N/A	Induce TAMs to M1	Female BALB/c mice	*In vivo* and vitro	(100)
PAMAM dendrimers	Glioma	~4	Fluorescent dye	N/A	Penetrate BBB and tumor ECM	Female Fischer 344 rats	*In vivo*	(101)
RBC	BC	N/A	Zoledronate	N/A	Deplete macrophages	Female Balb/C mice	*In vivo*	(102)
Sensitive cluster NPs	BC, CC, MEL	10- 90	Platinum BLZ-945	CSF-1R	Deplete TAMs	BALB/c mice	*In vivo* and vitro	(103)
SWNT	Glioma	N/A	CpG	TLR9/NF-κB	Inhibit metastasis	N/A	*In vivo* and vitro	(104)

AuNRs, gold nanorods; BBB, blood-brain barrier; BC, breast cancer; CC, colon cancer; CMC, carboxymethylcellulose; CNTs, carbon nanotubes; CNP, cationic nanoparticles; CPMV, cowpea mosaic virus; DSF/Cu, disulfiram/copper complex; ECM, extra cellular matrix; Exo-Ab, exosomes conjugated with antibodies; HDL, high-density lipoprotein; HS-PEG, mercapto polyethylene glycol with methoxy; LA, lactic acid; LC, lung cancer; LOX, lactate oxidase; MEL, melanoma; MpSi, mesoporous silicon; N/A, not available; Nab, nanoparticle albumin-bound formulation; NK-NPs, NK cell-membrane-cloaked NPs; PCA, pancreatic cancer; PDAC, pancreatic ductal adenocarcinoma; PTX, paclitaxel; Rego, regorafenib; RBCs, red blood cells; SWNT, single-walled carbon nanotubes; TAMs, tumor-associated macrophages; TCPP, 4,4’,4’’,4’’’-(porphine-5,10,15,20-tetrayl) tetrakis (benzoic acid); TLR, toll-like receptor.

Anti-cancer drugs engulfed in macrophages-loaded NPs can reduce toxicity and increase the loading ratio. Notably, the phagosomes of macrophages might cause degradation of the drugs and affect the functions of TAMs, thus there are accumulating studies aimed at addressing and solving these defects. For instance, in a mouse model of pulmonary metastases, Kim et al. developed the lipid–NPs conjugates and incubated them into the macrophage membrane to encourage the hydrophobic compound to conjugate with TAMs effectively and stably for anti-tumor therapy (106). Recently, genetic engineering, surface modification, and cellular backpacks have been able to modulate the function of macrophages, enhancing the infiltration of solid tumors. For example, as CRISPR-Cas9 gene-editing technology could influence the expression of surface markers, macrophages showed a four-fold increase in the elimination of cancer cells with arginine NPs (107). Through cell surface modification, hybridizing macrophages with NPs that provide numerous new sites for anti-cancer drug loading could decrease the toxic effect on macrophage carriers (108). Furthermore, polymer patches, as phagocytosis-resistant backpacks, conjugated to the surface of macrophages and crossed the BBB without changing the TAMs functions, including targeting and phagocytosis ability, and the properties of the loaded particles (109). In summary, these data suggest that modulating the contact area and enhancing bonding strength could further ensure stability when penetrating the solid tumor.

With a diameter of 40-160 nm and a specific content of RNA, proteins, and other compounds, macrophage-derived exosomes are derived from the invagination of the cellular membrane and processed into mature multivesicular bodies (MVBs) in the cytoplasm (110, 111). Exosomes, especially M1 macrophage-derived exosomes (M1-exos), inherit similar surface membrane properties from macrophages and can be employed to deliver various anti-tumor components *via* crosstalk between TAMs and cancer cells. For instance, Harney et al. found that in nude mice with triple negative breast cancer (TNBC) cells, M1-exos loaded with paclitaxel (PTX) or doxorubicin (DOX) could target cancer cells and enhance the anti-tumor effects (112). Since exosomes are a highly heterogeneous population of membrane vesicles and no specific biomarkers have yet been confirmed, the isolation and purification of M1-exos are challenging, and can be resolved by developing exosome-mimetic vesicles or proper genetic engineering. It is worth noting that THP-1-derived exosomes are sensitive to extracellular stimulation, including lipopolysaccharide (LPS) (113) and IL-4 (114), by modulating inherent exosomal contents, and have a relatively fixed size, which could influence the stability and selection of loading NPs.

Unlike exosomes, membrane-coated NPs without other contents are flexible in size and are unacted upon by the surrounding status, leading to an effective and stable therapeutic effect with a sufficient drug-loading capacity and nanosize. Purified macrophage membranes, obtained from disrupted cells by centrifugation, can coat NPs by a direct extrusion method, and have favorable biocompatibility and tumor-homing ability in systemic circulation. For instance, Cao et al. showed that using macrophage membranes to coat liposomes loaded with cytotoxic anti-cancer drugs could increase the cellular uptake of NPs and effectively inhibit lung metastasis, owing to the interaction between integrins on membranes and VCAM-1 on cancer cells, as well as CCR2 and CCL2 (115). However, since membrane vesicles without an intact signaling axis need to be engulfed by cancer cells for drug delivery, the membrane at this time inhibits NP release when accumulating in the TME. To support this, Zhang et al. found that modulating NP properties for effective membrane escape was necessary with pH-sensitive polymer, taking advantage of the pH difference between the TME and vesicles to influence the water influx, expansion, and eruption of membrane coating (116).

### Materials based on TAMs for tumor imaging

At present, TAM-based imaging has been performed in magnetic resonance imaging (MRI), fluorescent imaging, and positron emission tomography (PET) for research investigation in cancers (**Figure 2C**). Since the high-pressure properties of hypoxia TME restrict the effective aggregation and tissue penetration of materials targeting cancer cells directly (117), TAMs, which have high phagocytic ability and a wide distribution in tumor sites, can guarantee the relevant ingredients enrichment. In this case, by observing the difference in the number and phenotype of TAMs, TAM-based imaging can not only be useful for tumor diagnosis and prognosis, but also for addressing the edge of tumors for surgery. For instance, macrophage-specific radiotracers or probes can be designed as antibodies with TAM-specific biomarkers, folate (M1) and mannose receptor (M2), coupled to signal probes of magnetic resonance agents and radionuclides, or TAM-related NPs with signal-giving moieties (118), suggesting that using TAM-based materials to transmit signal compositions of imaging is a promising strategy.

MRI contrast agents (Cas), including gadolinium (Gd), iron oxide NPs, and fluorine 19 (^19^F), are critical in obtaining accurate contrast-enhanced anatomical images. Due to the rapid elimination of Gd through renal metabolism, Rayamajhi et al. found that macrophages could act as Gd carriers with cell-derived hybrid extracellular vesicles (Gd-HEVs) to prolong the retention time (119). As Gd exerts nephrogenic systemic fibrosis (NSF) toxicity, interestingly, intravenously injected iron oxide NPs are preferentially phagocyted by macrophages to decrease toxicity. For instance, ferumoxytol, a type of iron oxide NPs for macrophages, caused tumor enhancement on postcontrast scans by MRI image due to the existence of CD68 and CD163 TAMs (120). However, since iron oxide NPs can also be phagocyted by neutrophils and other phagocytic cells, the accuracy and specificity of these imaging techniques need to be improved. Notably, ^19^F, as perfluorocarbon compounds (PFCs), can be delivered intravenously in the same way as iron oxide NPs, and can image macrophages better due to its low background. To support this notion, Khurana et al. showed a strong association between the quantity of ^19^F atoms and the number of macrophages after PFC injection (121), and ^19^F accuracy is more reliable than ferumoxytol (122).

Fluorescence imaging can be used for cancer imaging by detecting TAMs. Using knock-in mice whose macrophages were marked with fluorescent dextran, various populations of myeloid-derived cells showed different biological behaviors and were distributed in different parts of the tumor (123), indicating that monitoring macrophages using fluorescence imaging is feasible. On account of the unique overexpression of folate receptor (FR)-*β* ^+^ in TAMs, folate-conjugated fluorescein isothiocyanate (folate-FITC) could be utilized for the precise optical detection of tumors by targeting M1 macrophages (124). More experiments are needed for conventional exogenous fluorophores before clinical applications. In addition, endogenous fluorescence metabolites, including nicotinamide adenine dinucleotide (NADH) and flavin adenine dinucleotide (FAD), involved in glycolysis and cellular respiration are sources of autofluorescence signals and can also be used to quantify TAMs. For instance, Szulczewski et al. discovered that macrophages accounted for almost 75% of the cells with elevated FAD inside a mammary cancer mouse model (125). In summary, since fluorescence imaging reports changes in TME longitudinally over time and reflects the immediate and divergent therapy effects, the half-time of materials influenced by the renal route of excretion should be fully considered.

Although PET can monitor a high enrichment of nanotracers non-invasively, limited data have supported that TAM-related NPs are good choices for cancer PET imaging. Different PET tracers might be valuable for monitoring TAM immunology by appropriately integrating with NPs. For instance, Pérez-Medina et al. performed an analysis of ^89^Zr-PL-high-density lipoprotein (HDL) and ^89^Zr-AI-HDL, which can be contained by TAMs and act as delivery cargo, to assess the burden of TAMs *in vivo* using PET imaging (126). However, HDL also targets other immune cells (127), indicating that the accuracy of TAM-related NPs in PET imaging needs to be further explored. Taken together, the evidence suggests that TAM-based tracers in MRI, fluorescence imaging, and PET highlight potential avenues for cancer diagnosis and therapy. Despite focusing on TAMs themselves, designing materials to detect TAM-derived secretions might be a potential orientation of imaging. In an alternative approach, combining MRI with fluorescence imaging or PET for particular macrophage polarizations might increase the precision of MRI based on TAMs.

## Materials based on tumor-associated macrophages for targeting immune cells

**Figure 3:**
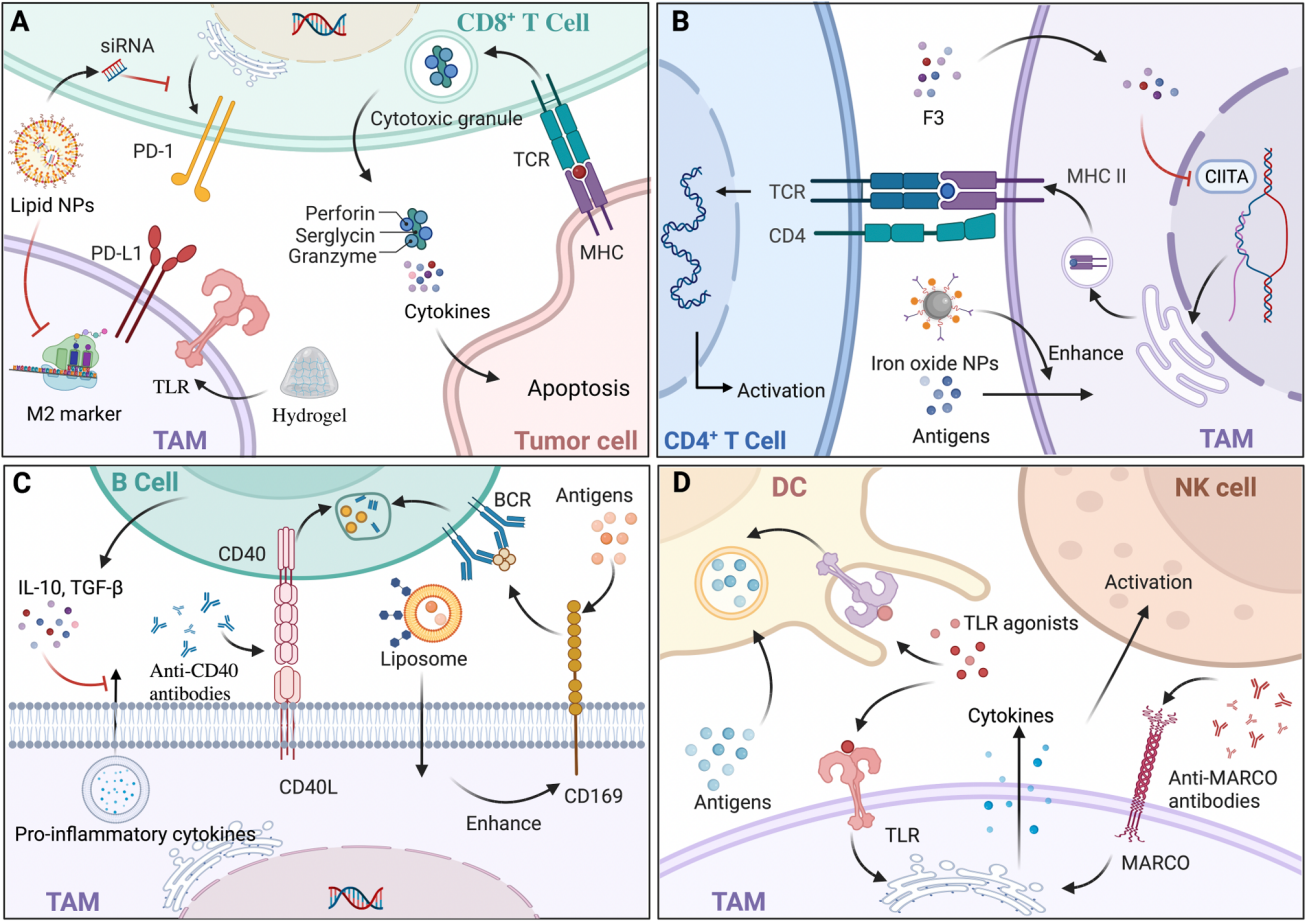
The interaction and materials based on tumor-associated macrophages (TAMs) to target immune cells. **(A)** CD8^+^ T cells induce tumor cell apoptosis mainly through the release of cytokines and the exocytosis of granules containing perforin and granzyme. Hydrogel, a type of nanoparticle (NP), loaded with antigens can activate CD8^+^ T cells indirectly with the help of macrophages expressing toll-like receptors (TLRs). In addition, lipid NPs loaded with siRNA could specifically block the M2-marker expression of TAMs and lead to decreased expression of PD-1. **(B)** TAMs expressing major histocompatibility complex class II (MHC-II) present tumor antigens and activate CD4^+^ T cells with T cell receptor (TCR). There are two ways to fully boost CD4^+^ T cells, one of which is modulating MHC-II expression by epigenetic silencing with chromatin modifiers, including *S. crispus* leaves (F3), that can inhibit CIITA transcription; the other is enhancing antigens presentation and MHC-II pathway by iron oxide NPs. **(C)** Regulatory B (Breg) cells secrete interleukin‐10 (IL‐10) and transforming growth factor‐β (TGF‐β), decreasing pro-inflammatory cytokine secretion of TAMs, which modulate the biological behavior of tumors. Since CD169^+^ macrophages capture antigens and present to B cell receptor (BCR) on B cells, liposomal NPs with glycan ligands were preferentially phagocytized to modulate B cells indirectly. Anti-CD40 antibodies acted on CD40-CD40L pathways with a greater upregulation of anti-tumor activity. **(D)** TAMs transfer antigens to dendritic cells (DCs) by CD169 for immunotherapy. Since TLRs exist on both DCs and TAMs, TLR agonists could enhance antigens presentation of DCs and pro-inflammation cytokines’ secretion of TAMs, leading to the immunostimulatory tumor microenvironment (TME). *Via* the increase of cytokines, such as IL-15, anti-MARCO antibodies activate natural killer (NK) cells. Back arrows: promotion; Red “T” arrows: inhibition.

Except for cancer cells and TAMs, the TME is composed of fibroblasts, endothelial cells, various types of immune cells, etc. In addition, adaptive immune cells (T cells and B cells) and innate immune cells, including macrophages, neutrophils, dendritic cells (DC), and natural killer (NK) cells, also influence the occurrence and development of tumors. Participating in innate and adaptive immunity, macrophages, as mediators, play an important role in regulating the immune response to tumors (**Figure 3**).

### CD8^+^ T cells and their application in TAM-related materials

The crosstalk between CD8^+^ T cells and macrophages undoubtedly exacerbates the immunological escape of cancer cells and has a negative effect on cancer immunotherapy. CD8^+^ T cells differentiate into cytotoxic T lymphocytes (CTLs) and CTLs exert anti-tumor functions in three ways: physical contact with cancer cells *via* intracellular signal activation, the release of cytokines, and exocytosis of granules containing perforin and granzyme (128). By stimulation of TNF‐α and IFN‐γ secreted from M1 macrophages, CD8^+^ T cells transformed into a cytotoxic phenotype with upregulated PD-1 expression (129). In contrast, influenced by IL-10 and TGF‐β secreted by M2 macrophages, CD8^+^ T cells were kept away from cancer cells and their immune activity was attenuated, resulting in the immunological escape of cancer cells (130). Thus, TAM-related materials including microparticles (MPs), NPs, and RNAs could be equipped with the ability to impact the infiltration of CD8^+^ T cells to affect anti-tumor immunity (**Figure 3A**). Mannose-modified macrophage-derived MPs equipped with metformin (Met@Man-MPs) can polarize TAMs to M1 phenotype and remodel the TME by recruiting CTLs through the degradation of collagen fiber (131). While MPs with a particle size of 100-1000 nm limited tissue biodistribution, it is suggested that developing MPs with a narrower size or nanosize would be ideal for the delivery of drugs. To support this, Muraoka et al. found that NPs could activate CD8^+^ T cells indirectly through the TLRs expressed by macrophages (132). In another study, conjugated with recombinant ovalbumin (OVA), gold NPs with a diameter of 10-22 nm, which were engulfed by macrophages, exhibited greater anti-tumor efficacy with the further infiltration of CD8^+^ T cells (133), indicating that the immune response of NPs would also be mediated by particle size. Additionally, TAMs targeting NPs with RNAs would decrease T cells apoptosis and enhance CTL activation by either influencing the IL-6 and IL-10 secretion of TAMs (134) or modulating the amount of diverse TAM phenotypes causing CTL infiltration (46). Notably, challenges still exist regarding NPs with RNAs for delivery blockade in terms of digestion by various enzymes, unreachability to target cells, and endocytosis before proper degradation. To sum up, since CD8^+^ T cells exert tumor elimination by a relatively direct method, TAM-related materials as drug adjuvants or carriers for the further infiltration of CD8^+^ T cells have great potential to ensure essential contact with cancer cells.

### CD4^+^ T cells and their application in TAM-related materials

CD4^+^ T cells differentiate into various subtypes to participate in immunity, among which T-helper 1 (Th1) and Th2 account for the majority. Considered as the most essential helper cell type for cancer immunity, Th1 cells eliminate cancer cells indirectly with the help of MHC-II positive macrophages (135), and Th2 cells exert anti-tumor functions by inducing inflammation in TME with the participation of M2 macrophages (136). Given that TAMs in TME act as APCs expressing MHC-II to present tumor antigens, TAM-related materials are promising in activating CD4^+^ T cells (**Figure 3B**). For instance, Rong et al. developed iron-chelated melanin-like NPs (Fe@PDA-PEG) to promote TAM polarization and the recruitment of Th cells by high efficacy in capture, phago-endocytosis, processing, and the presentation of tumor-associated antigens (TAAs), subsequently leading to suppressed tumor progression (137). As MHC-II expression can be influenced by its transactivator (CIITA) (138), it indicates that materials targeting CIITA transcriptional activity is potential by affecting promoter selection, mRNA stability, and post-translational modification. For example, Yankuzo et al. discovered a bioactivator of *S. crispus* leaves (F3) by facilitating IFN-γ production in the tumor site, which could induce CD4^+^ T cell infiltration and increase CIITA and MHC-II expression of cancer cells with the attenuation of CD68^+^ TAMs (139). Notably, without the interaction between CD4^+^ T cells and TAMs, IFN-γ itself could not modulate TAMs for tumor elimination (140), indicating that anti-tumor materials based on cytokines should fully consider the crosstalk among immune cells and can be integrated with specific agents to mimic the essential signaling pathways. In summary, the evidence suggests that TAM-related materials could be conducted by epigenetic modulations targeting MHC-II and accessory genes to guarantee antigen presentation to CD4^+^ T cells efficiently and to increase the abundance of CD4^+^ T cells for sufficient interaction with macrophages.

### B cells and their application in TAM-related materials

Tumor-infiltrating B lymphocytes (TIBs) with a strong capacity for immune responses are crucial in the TME for activating T cell responses and causing cancer cell death, while regulatory B (Breg) cells decrease the pro-inflammatory cytokine secretion of macrophages and promote tumor progression (141) (**Figure 3C**). In inflammatory tumor tissue, CD169^+^ macrophages, which capture intact antigens and preserve on their surface for a long time, cross-present TAAs to TIBs, and in turn, B cells secrete neurotransmitter GABA (γ-aminobutyric acid), which could increase the ratio of anti-inflammatory macrophages (142). For the sake of convenience in further exploring the interactions between TAMs and B cells, peritoneal cavity (PerC) cell culture serves as an ideal *in vitro* system, as it concludes most B cell subtypes (143). At present, materials that modulate B cells mainly act on either macrophage, which could affect TIB indirectly or the signaling pathways between them (**Figure 3C**). For instance, Chen et al. showed that newly designed liposomal NPs with glycan ligands, which were affiliative to CD169, could effectively deliver antigens to CD169^+^ macrophages (144), modulating B cell biological behavior indirectly. While it is remarkable that using glycan is lacking in specificity, developing ligands targeting an exclusive receptor might enhance anti-tumor therapy. Regarding signaling pathways, in preclinical studies, anti-CD40 IgG1 mcAbs exerted strong anti-tumor activity with a greater upregulation of activation markers on B cells by modulating CD40-CD40L interaction between macrophages and B cells, leading to higher efficacy of antigen presentation and immune response (145). Similarly, Bruhns et al. found that IgG1 was more appropriate for immunostimulatory activities compared with the other IgG antibodies (146). Further, since FcγRIIB is the only FcγR on B cells, upregulation of FcγRIIB might improve the efficacy of the specific mcAbs. Thus, materials aiming at gene modification have potential by influencing receptor expression or lipid rafts incorporation for the essential distribution of FcγRIIB.

### DCs and NK cells and their application in TAM-related materials

DCs exert anti-tumor effects *via* TAA uptake, processing, and presentation, which display an extensive dysfunctional status under the influence of TAMs secreting immunosuppressive factors (147). To solve the numerical and functional DC deficiencies, TAM-related materials, which can promote DC activation and reverse the immunosuppressive TME, are potential treatment modalities (**Figure 3D**). For instance, TLR agonists could act as therapeutic materials, since TLR can not only stimulate DC maturation, antigen presentation, and cytotoxicity towards tumor cells (148), but also activate M1 macrophages to secrete pro-inflammation cytokines and create a potent immunostimulatory microenvironment (149). To date, three TLR agonists have been developed for tumor therapy in a clinical setting: imiquimod as a TLR7 agonist for the treatment of superficial basal cell carcinoma, Bacillus Calmette-Guérin (BCG) as a TLR2, TLR3 and TLR9 agonist for non-invasive transitional cell carcinoma of bladder therapy, and monophosphoryl lipid A (MPLA) as a TLR4 agonist for the human papillomavirus (HPV) vaccine to prevent cervical cancer (150). Of note, since DCs would rather phagocytize particles with a diameter of 20–200 nm (151), the drug size should be taken into consideration for the design of materials. Additionally, NK cells exhibiting a quick response to infections and malignancies are crucial in innate immunity, and can be activated by M1 macrophages secreting IL-12, and suppressed by M2 subsets secreting TGF-β (152). Similar to DCs, TAM-based materials act on NK cells by modulating related cytokines to mediate immunosuppression in the TME. For instance, Eisinger et al. found that performing an anti-MARCO antibody on TAMs could activate NK cells to inhibit tumor growth by modulating the IL-15 secretion of macrophages, a cytokine known to support NK cell proliferation, infiltration, and cytotoxic capacity (153). In addition, as macrophage subtypes have their own distinct effects on NK cells, materials targeting TAM polarization could indirectly modulate the biological behaviors of NK cells and cytokines in the TME (154). Together, compared with the materials acting on DCs and NK cells directly which might induce possible excessive immune activation because of targeting DCs and NK cells without specificity of distribution (155), and lead to off-target and normal tissue injury (156), TAM-related biomaterials would reverse the immunosuppressive TME and restore normal immunity to exert more effective anti-tumor functions.

## Conclusions

In this review, we conclude the specific properties and various effects of TAM-based materials from bench to clinic. Targeting diverse crosstalk among TAMs, cancer cells, and immune cells, a variety of biomaterials with TAMs as accessory cells or carriers could modulate TAMs biological behaviors, exert tumor elimination and imaging, and engage adaptive and innate immunity for anti-tumor therapy. However, there are some limitations of these materials that need to be overcome.

Firstly, highly heterogeneous macrophages exhibit various immune responses to TAM-related materials of deficient specificity, which can easily lead to off-target and unstable anti-tumor effects. Hence, it is necessary to further investigate TAMs subtypes differentiation, design specific targeting molecules and develop more efficient tumor diagnostic and therapeutic modalities.

Secondly, since most research on biomaterials effects is conducted *in vivo*, data from mouse models require expanded studies to verify accuracy and biocompatibility for clinical application. It would be feasible to define a ‘TAM atlas’ of human solid tumors and compare it with mice data using emerging technologies, including tissue mass spectrometry, single-cell RNA sequencing (scRNA-Seq), and multiplex immunofluorescence, to predict immunotherapy treatment prognosis (157).

Thirdly, therapeutic strategies targeting macrophages might have potential adverse reactions. For instance, CSF-1R inhibitors can reduce the number of TAMs *in vivo*, but unexpectedly recruit a large number of immunosuppressive granulocytes (158). Furthermore, depletion of CD169^+^ macrophages can inhibit tumor growth and increase the infiltration of CD8^+^ T cells in the TME, unfortunately doing harm to bone homeostasis and bone marrow erythropoiesis (159). Thus, TAM-targeted therapy needs to further explore adverse reactions and the mechanisms of the drugs, possibly combining with other agents to reduce systemic toxicity.

## Author contributions

QG and FW conceived and designed the study. FJ drafted the manuscript. FJ, XL, XC and FW searched and reviewed the literatures, and made the figures and tables. All of the authors critically reviewed and revised the manuscript. All authors have read and agreed to the published version of the manuscript.

## Funding

This research was funded by the National Natural Science Foundation of China (No. 82002884), the National Key Research and Development Program of China (No. 2020YFA0714001), Sichuan Science and Technology Program (No. 2015JY0046), Science and Technology Program of Chengdu City (No. 2021-YF05-02031-SN) and the Undergraduate Innovation and Entrepreneurship Training Program of Sichuan University (No. 2022120939).

## Acknowledgments

A part of the figures were created with BioRender.com. Due to space limitations, we apologize for not being able to cite and discuss all relevant work from different laboratories published to date.

## Conflict of interest

The authors declare that the research was conducted in the absence of any commercial or financial relationships that could be construed as a potential conflict of interest.

## Publisher’s note

All claims expressed in this article are solely those of the authors and do not necessarily represent those of their affiliated organizations, or those of the publisher, the editors and the reviewers. Any product that may be evaluated in this article, or claim that may be made by its manufacturer, is not guaranteed or endorsed by the publisher.
